# Supplementary material for: Influence of Five Drying Methods on Active Compound Contents and Bioactivities of Fresh Flowers from Syringa pubescens Turcz
Source: Molecules. 2023 Nov 27;28(23):7803. doi: 10.3390/molecules28237803 (PMC10708128; doi:10.3390/molecules28237803)
Supplement: Supplementary file 1 [file molecules-28-07803-s001.zip › molecules-2678559-supplementary.pdf]

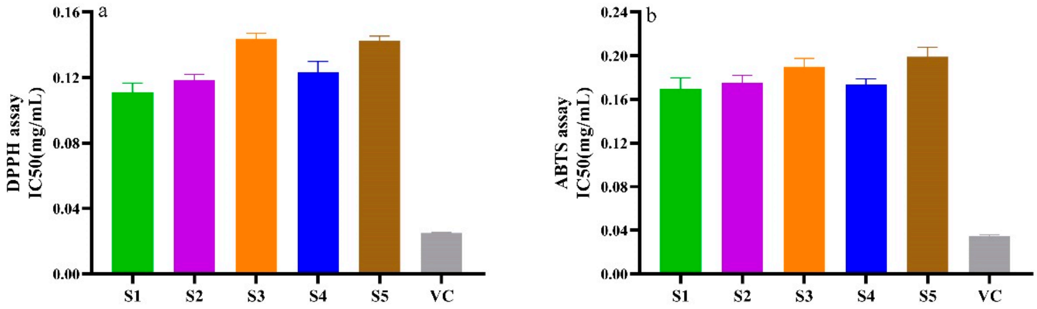

Figure. S1. IC_{50} values of SPF samples. (**a**) DPPH free radical; (**b**) $ABTS^{+}$ free radical;

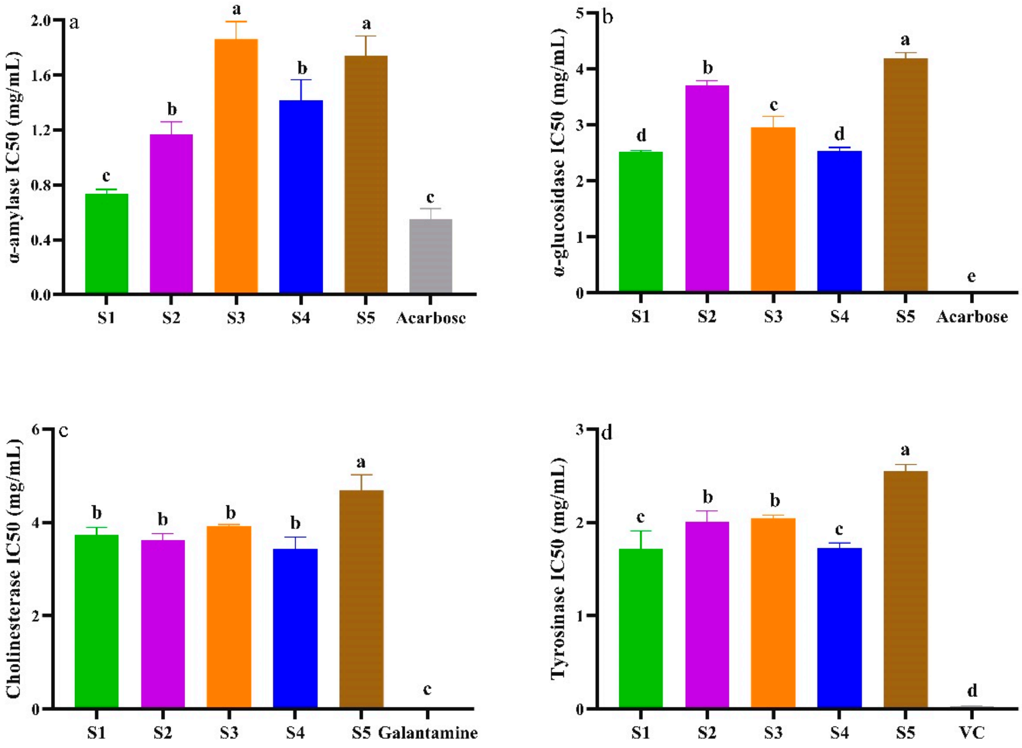

Figure. S2. IC_{50} values of enzyme inhibition assay. (a) α-amylase inhibitory activity of SPF; (b) α-glucosidase inhibitory activity of SPF; (c) AChE inhibitory activity of SPF; (d) Tyrosinase inhibitory activity of SPF.
